# Corneal Safety and Stability in Cases of Small Incision Lenticule Extraction with Collagen Cross-Linking (SMILE Xtra)

**DOI:** 10.1155/2019/6808062

**Published:** 2019-04-14

**Authors:** Ihab Mohamed Osman, Hany Ahmed Helaly, Mohsen Abou Shousha, Amir AbouSamra, Islam Ahmed

**Affiliations:** Ophthalmology Department, Faculty of Medicine, Alexandria University, Alexandria, Egypt

## Abstract

**Purpose:**

To assess the safety and stability in cases of small incision lenticule extraction with collagen cross-linking (SMILE Xtra).

**Methods:**

This study was a retrospective interventional comparative study that included 60 eyes of 30 patients divided equally into two groups: SMILE Xtra and SMILE alone. The inclusion criteria were patients >18 years of age, myopic error >6 D, thinner cornea <520 microns, and abnormal corneal topography. Outcome data were recorded including uncorrected distance visual acuity and corrected distance visual acuity (UDVA and CDVA), manifest refraction spherical equivalent (MRSE), central corneal thickness, average keratometry, endothelial cell density, corneal resistance factor (CRF), and corneal densitometry. The follow-up period was 24 months.

**Results:**

There was a significant difference between the 2 groups regarding UDVA, CDVA, and MRSE at 1 month. In the SMILE Xtra group, 90% of eyes had postoperative UDVA of 20/20 and 97% had UDVA of 20/30 at 24 months. At 24 months, 26 eyes (87%) vs. 25 eyes (84%) were within ±0.50 D of attempted correction in SMILE Xtra and SMILE groups, respectively. Regarding stability, both groups showed improvement of MRSE at 1^st^ month postoperatively and remained stable along the 24 months of follow-up. CRF and corneal densitometry were higher in the SMILE Xtra group along the whole follow-up period (*p*=0.001).

**Conclusion:**

Combining corneal cross-linking with SMILE procedure (SMILE Xtra) is a promising tool to prevent ectasia in high-risk patients. It is a safe and simple procedure that can be offered to patients undergoing SMILE with risk for ectasia. Trial registration no: PACTR201810577524718.

## 1. Introduction

Small incision lenticule extraction (SMILE) is a refractive procedure that is used to treat myopia and myopic astigmatism in a safe and effective way. It is the third-generation refractive laser vision correction procedure after first-generation photorefractive keratectomy (PRK) and second-generation laser in situ keratomileusis (LASIK) [1–4]. SMILE has the advantage over LASIK in being a flapless procedure. SMILE avoids flap-related complications, has potentially better biomechanical stability, and induces less postoperative dry eye [5–8].

One of the most feared complications after laser vision correction is corneal ectasia. In the early stages of ectasia, the eye can be treated with corneal collagen cross-linking (CXL). However, in more advanced cases, the definitive treatment is a keratoplasty procedure [9–11]. Risk factors for post-LASIK ectasia include missed forme fruste keratoconus and factors leading to a thin residual stromal bed thickness such as thin preoperative central corneal thickness and high myopic error [12, 13]. In cases expected to develop post-LASIK ectasia, simultaneous use of CXL in the same sitting was done to strengthen the residual cornea after ablation which is known as LASIK Xtra procedure [14–17].

Even though SMILE offers better biomechanical stability for the cornea, there are reports of cases of post-SMILE ectasia in the literature [18–22]. A simultaneous combination of SMILE procedure and CXL (SMILE Xtra) was prescribed to further strengthen the cornea in doubtful cases to decrease the risk of postoperative ectasia and may theoretically decrease the myopic regression [23–25].

The aim of the current study was to assess the safety and stability in cases of small incision lenticule extraction with collagen cross-linking (SMILE Xtra).

## 2. Subjects and Methods

This study was a retrospective interventional comparative study that included 60 eyes of 30 patients. The inclusion criteria were patients >18 years of age, myopic error of >6 D, thinner cornea <520 microns, and abnormal corneal topography. The included eyes were divided into two groups. The first group included 30 eyes of 15 patients that had undergone SMILE Xtra procedure. The second group included 30 eyes of 15 patients, that had undergone SMILE procedure alone, to act as a control group. The patients included in the SMILE group had the same criteria of selection as the other group but due to a financial reason or patient refusal had undergone SMILE only without CXL. Exclusion criteria for both groups included associated corneal pathology, established keratoconus, corneal thickness <450 microns, and hyperopia or mixed astigmatism.

The ethics committee of the Faculty of Medicine, Alexandria University, Egypt, approved this study. The guidelines of the Declaration of Helsinki were adhered to. The patients of the current study signed an informed consent before the intervention.

All included patients had preoperative complete ophthalmic clinical examination including slit-lamp examination, intraocular pressure measurement, fundus examination, cycloplegic refraction, uncorrected distance visual acuity (UDVA), and corrected distance visual acuity (CDVA). Corneal topography was done using a Wavelight topolyzer VARIO diagnostic device (Alcon Laboratories, Inc., Fort Worth, TX). The Pentacam Scheimpflug system (Oculus, Inc., Wetzlar, Germany) was used before the procedure to measure objectively the corneal densitometry values. The Scheimpflug system quantifies the density of the cornea on a scale from 0 to 100. Peak densitometry values were recorded directly from the axis line appearing in the Scheimpflug image. The axis nearest to the maximum K reading was determined, and the Scheimpflug image at the nearest meridian to this axis was used for analysis. Data recorded included central corneal thickness (CCT) and average keratometry readings. Corneal endothelial cell density (ECD) (in cells/mm^2^) was measured by using noncontact specular microscopy (Tomey EM-3000, Tomey Corporation, Nagoya, Japan). The ocular response analyzer (ORA; Reichert Corporation; Depew, USA) was used to measure corneal resistance factor (CRF) which is a measure of the cumulative effects of both the viscous damping and elastic resistance of the cornea [26]. The average of 3 good quality scans was used for all the previous measurements.

### 2.1. Surgical Technique

All surgeries were performed by a single surgeon (IO) using the same reproducible technique. The SMILE procedure was done under topical anesthesia using the VisuMax® 500 kHz femtolaser system (Carl Zeiss Meditec, Jena, Germany). The refractive target was to achieve emmetropia. For lamellar cuts, a spot distance of 3 *μ*m was used and for the side cuts and spot distance was 2 *μ*m. The spot energy was adjusted to a hundred and thirty nanojoules in the included patients. The minimum lenticule side cut thickness was adjusted to 10 *μ*m. The lenticule side cut angle was set to 130°, the incision side cut angle was set to 70°, and the lenticule diameter was set to 6.5 mm. The cone used was the small-sized one. The cap diameter was 7.5 mm. An intended cap thickness of 90 microns was used in all patients. After femtolaser application, a blunt spatula was used to loosen the stromal lenticule, and then it was removed using forceps. For the SMILE Xtra group, the corneal cross-linking procedure was done using CXL-365 vario system (Schwind, Germany). The procedure began with instilling a mixture of 0.1% riboflavin (vitamin B2) and 20% dextran 500 (MedioCROSS D, Peschke Meditrade GmbH, Huenenberg, Switzerland) into the corneal pocket through the SMILE incision for 15 minutes. Slit-lamp examination was done to confirm complete riboflavin absorption through the stroma and into the anterior chamber. Then, the machine was turned on to irradiate ultraviolet-A (UV-A) of 365 nm wavelength for 3 minutes at an irradiance of 18 mW/cm^2^ (total energy: 3.2 J/cm^2^). Then, the stromal pocket was flushed with saline. No intraoperative complications were recorded.

Postoperative treatment was in the form of topical antibiotic and steroids 4 times daily for 1 week, and then steroids eye drops was tapered over 1 month. Patients used preservative-free artificial tears for 2-3 months. Records of the follow-up visits at 1^st^ day, 1^st^ week, and 1^st^, 3^rd^, 6^th^, and 12^th^ months were reviewed. Patients were recalled for a final follow-up visit at the 24^th^ month postoperative. Outcome data were recorded including UDVA, CDVA, manifest refraction, CCT, average keratometry, ECD, CRF, and corneal densitometry. Efficacy index (the ratio of postoperative UDVA to preoperative CDVA) and safety index (the ratio of postoperative CDVA to preoperative CDVA) were calculated. Predictability and stability of refractive correction were recorded. Any complication was also recorded.

Data analysis was performed using the software SPSS for Windows version 20.0 (SPSS Inc., Chicago, USA). Quantitative data were described using range, mean, and standard deviation. The independent-samples *t*-test was used to compare means of different samples. The paired *t*-test was used for comparisons between means of the preoperative and postoperative data. Linear regression was used to correlate between the attempted and achieved spherical equivalent. The chi-squared test was used to compare between different percentages and ratios. Differences were considered statistically significant when the associated *p* value was less than 0.05.

## 3. Results

Both SMILE Xtra and SMILE groups included 30 eyes of 15 patients.  Table 1 shows the preoperative characteristics of included eyes of both groups. There was no statistically significant difference between the included patients of the 2 groups regarding the age and gender (*p* > 0.05). Eyes of the SMILE Xtra group had thinner and steeper corneas with more densitometry readings than that of the SMILE group, but this was not statistically significant using independent-samples *t*-test (*p* > 0.05).

**Figure 1:**
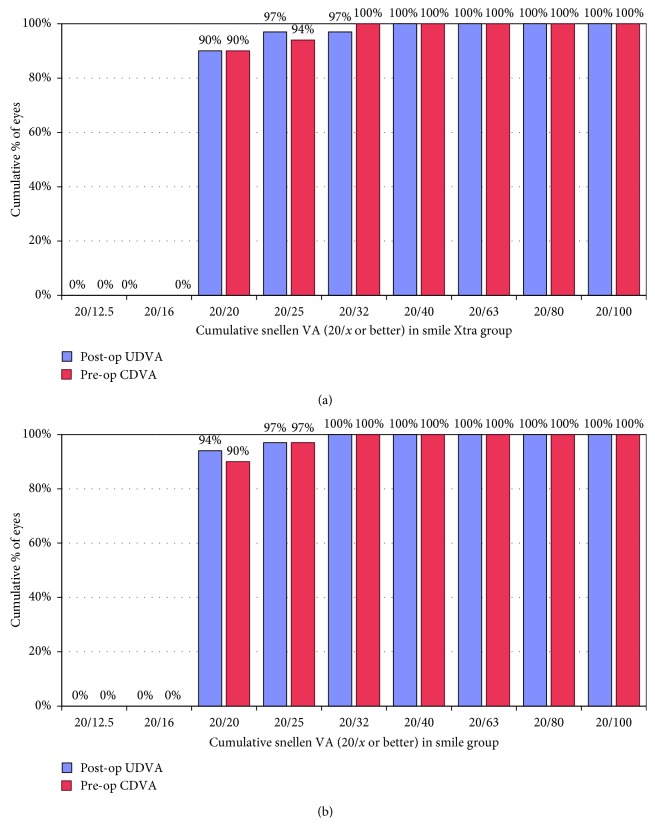
Cumulative Snellen visual acuity in (a) SMILE Xtra and (b) SMILE groups at 24 months.

**Figure 2:**
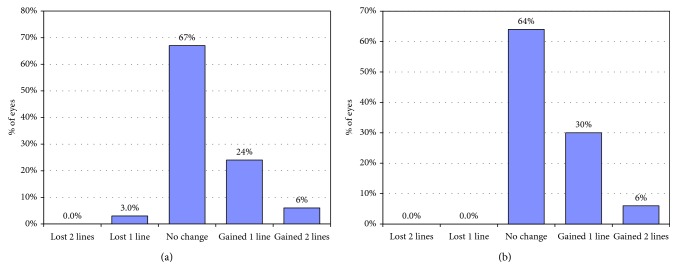
Changes in Snellen line in corrected distance visual acuity in (a) SMILE Xtra group and (b) SMILE group at 24 months.

Table 2 shows the postoperative visual acuity (UDVA and CDVA) and the residual manifest refraction spherical equivalent (MRSE) along the follow-up period. Using independent-samples *t*-test, there was a statistically significant difference between the 2 groups regarding UDVA, CDVA, and MRSE at 1 month. This difference became statistically nonsignificant at 6, 12, and 24 months. Using the paired *t*-test to compare the mean UDVA, CDVA, and MRSE at 1 month vs. 24 months, all values showed statistically significant difference (*p* < 0.05). In the SMILE Xtra group, 90% of eyes had postoperative UDVA of 20/20 and 97% had UDVA of 20/30 at 24 months. In the SMILE group, 94% of eyes had postoperative UDVA of 20/20 and 100% had UDVA of 20/30 at 24 months (Figure 1). At final follow-up, only one eye from the SMILE Xtra group lost 1 line while 2 eyes from both groups gained 2 lines (Figure 2). The overall mean efficacy index (postoperative UDVA/preoperative CDVA) at 24 months was 1.09 and 1.12 for SMILE Xtra and SMILE groups, respectively. The overall mean safety index (postoperative CDVA/preoperative CDVA) at 24 months was 1.29 and 1.28 for SMILE Xtra and SMILE groups, respectively.

**Figure 3:**
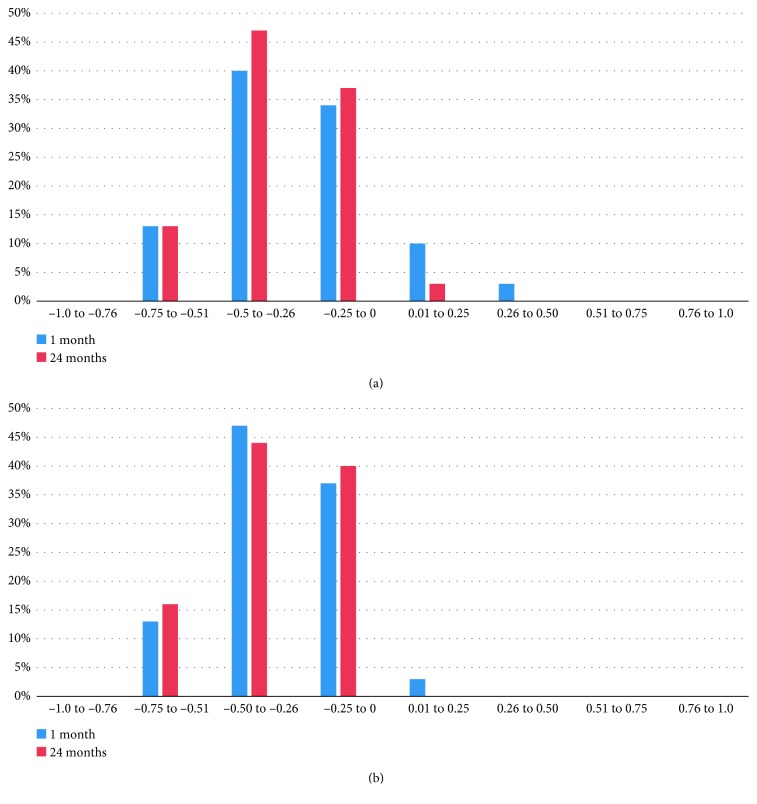
Distribution of postoperative spherical equivalent (predictability) among (a) SMILE Xtra group and (b) SMILE group at 1 and 24 months (*y*-axis: % of eyes; *x*-axis: spherical equivalent in diopters).

**Figure 4:**
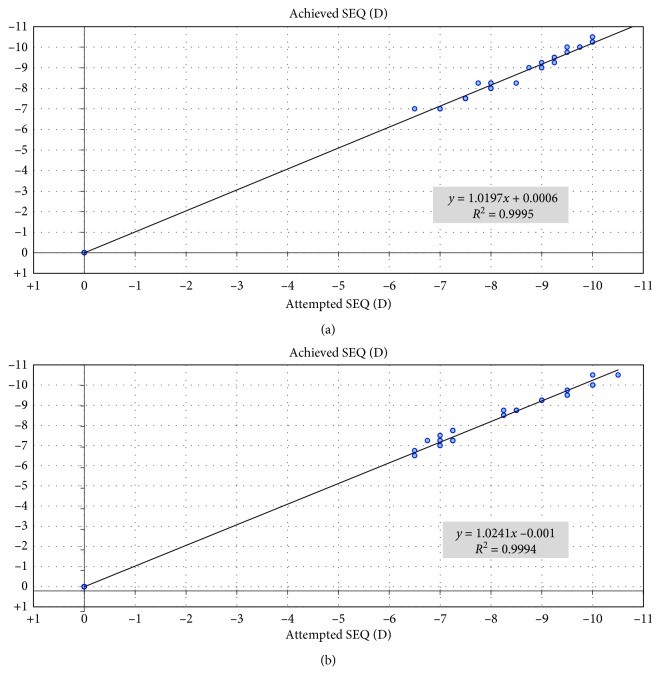
Attempted versus achieved manifest refraction spherical equivalent (SEQ) in (a) SMILE XTRA group and (b) SMILE group at 24 months.

**Figure 5:**
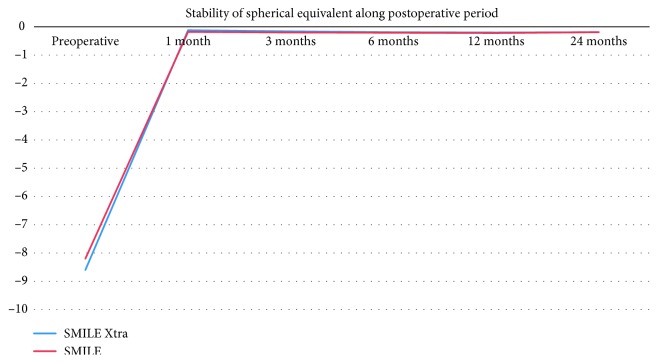
Stability of manifest refraction spherical equivalent over time in SMILE Xtra group and SMILE group (*y*-axis: spherical equivalent in diopters; *x*-axis: postoperative time interval in months).

Regarding predictability, both groups showed high levels of predictability. In SMILE Xtra and SMILE groups, 26 eyes (87%) were within ± 0.50 D of attempted correction at 1 month and 30 eyes (100%) were within ± 1.00 D at 1 month. At 24 months, 26 eyes (87%) vs. 25 eyes (84%) were within ± 0.50 D of attempted correction and 30 eyes (100%) vs. 30 eyes (100%) were within ± 1.00 D in SMILE Xtra and SMILE groups, respectively (Figures 3 and 4). Regarding stability, both groups showed improvement of MRSE at 1^st^ month postoperative and remained stable along the 24 months of follow-up (Figure 5). In the SMILE Xtra group, mean MRSE improved from preoperative levels of −8.6 ± 1.1 D to −0.12 ± 0.23 at 1 month and −0.18 ± 0.19 at 24 months. In the SMILE group, mean MRSE improved from preoperative levels of −8.2 ± 1.2 D to −0.18 ± 0.19 at 1 month and −0.19 ± 0.18 at 24 months (Table 2).

Table 3 shows average keratometry readings, central corneal thickness, and endothelial cell count along the postoperative follow-up period. Using the paired *t*-test to compare values at 1 month versus 24 months, there was no statistically significant difference (*p* > 0.05). Mean CCT of the SMILE Xtra group was statistically significantly thinner than that of the SMILE group at 6 and 24 months. Using the paired *t*-test to compare values at 24 months versus preoperative levels, mean CCT and K readings were statistically significantly different (*p* < 0.001), while mean ECD was not statistically significantly different (*p*=0.344, 0.467 in both groups, respectively).

Corneal resistance factor was significantly higher in the SMILE Xtra group along the whole follow-up period (*p*=0.001) (Table 4). Both SMILE Xtra and SMILE groups showed significant drop in CRF from preoperative levels (*p*=0.001). Corneal densitometry was significantly higher in the SMILE Xtra group along the whole follow-up period (*p*=0.001) (Table 4). Corneal densitometry increased significantly at the 1^st^ month and started to decrease over the next 24 months but did not reach the base line levels. The difference between preoperative and 24 months' levels for the SMILE Xtra group was statistically significant (*p*=0.001). The difference between preoperative and 24 months' levels for the SMILE group was not statistically significant (*p*=0.312).

## 4. Discussion

SMILE has the theoretical advantage of producing less weakening effect on the cornea as it is a flapless procedure with the cap thickness contributing to the residual bed thickness. SMILE also is considered a more tissue saving procedure when compared to LASIK (it takes less tissue per diopter 13 microns vs. 17 microns in LASIK). This makes SMILE a more suitable option in treating higher myopic errors, thinner corneas, and cases with abnormal topography or forme fruste keratoconus [23, 27]. However, as mentioned above, there are reports for corneal ectasia after SMILE [18–22].

Any corneal laser refractive procedure would certainly decrease the biomechanical stability of the cornea. The need for strengthening the cornea after a laser refractive procedure, especially in high-risk patients, seems logical. Combining corneal collagen cross-linking with PRK or LASIK showed good results and has come into practice, what is known as LASIK Xtra [28, 29]. In 2009, Kanellopoulos [30] published a study in which CXL was done using a femtosecond laser which created corneal pocket for cases of early keratoconus. He suggested an alternative to the conventional CXL with the advantage of not removing the epithelium thus having faster healing, better comfort, and less chance of infection. Combining corneal collagen cross-linking with SMILE procedure (SMILE Xtra) has used the same idea. Early published results of SMILE Xtra shows the procedure to be safe and effective on the short-term period. There are few articles in the literature covering this procedure, and all of them lack-long term follow-up [23–25, 31]. One advantage of SMILE Xtra is that the CXL is done to both the overlying cap and the underlying stroma; where in LASIK Xtra, it is done only to the underlying stroma as it may cause flap wrinkling and difficulty in relifting the flap for touch up.

Treating high-risk corneas with CXL as prophylaxis must be different from therapeutic protocols. The aim is to deliver the least amount of energy that can stabilize the cornea. Too much energy would cause haze and interfere with vision, while too little energy would be insufficient to provide the required corneal strength. For treating keratoconus, there are established CXL protocols with long-term follow-up that proved to be safe and effective [32]. However, for prophylaxis purpose to treat eyes at risk, there is no standard protocol yet with different suggested regimens. In LASIK Xtra procedure, different authors have used 30 mW/cm^2^ for different durations with a total energy of 1.8 to 5.4 J/cm^2^, and all those different regimens proved to be safe and effective [23, 29, 33]. Unfortunately, for prophylactic treatment, the minimum amount of energy to make the cornea strong enough is still unknown.

Our protocol for CXL during the SMILE Xtra procedure was to irradiate UV-A of 365 nm wavelength for 3 minutes at an irradiance of 18 mW/cm^2^ (total energy: 3.2 J/cm^2^) using the CXL-365 vario system (Schwind, Germany). Three minutes was roughly around half the duration we used for conventional keratoconus treatment. This amount of energy proved to be safe and well tolerated as none of the cases suffered from significant haze or any other serious complication (e.g., epithelial defects, deep lamellar keratitis, or punctate keratitis). Ganesh et al. [23] reported good safety outcomes at 12 months for the use of the Avedro KXL system (Waltham, MA) for accelerated cross-linking with UV-A radiation at 365 m wavelength for eyes at risk of ectasia, with energy of 45 mW/cm^2^ delivered in continuous mode for 75 seconds. Total energy delivered was 3.4 J/cm^2^. Ng et al. [24] used the CXL-365 vario system (Schwind, Germany) to deliver UV-A irradiation at 18 mW/cm^2^ for 45 seconds (total energy: 0.8 J/cm^2^) which we believe is too little amount of delivered energy. The authors defended the use of the shorter time by stating that they found around 10% of cases still having observable haze at 6 months with the use of longer time of treatment (unpublished data). Graue-Hernandez et al. [31] reported good refractive outcomes at 24 months for SMILE Xtra on eyes with forme fruste keratoconus by applying the standard Dresden protocol with 5.4 J/cm^2^ total energy. They used the UV-X device (UV-X 1000, IROC) to deliver UV-A of 370-nm wavelength with energy of 3.0 mW/cm^2^ for 30 minutes.

Both SMILE Xtra and SMILE groups in our study showed excellent efficacy and safety that remained stable along the 24 months of follow-up. At 1 month, UDVA and CDVA were significantly better in the SMILE group. This may be explained by the presence of mild haze in cases of SMILE Xtra which resulted in higher corneal densitometry using Scheimpflug imaging. At 3 months, there was no clinically significant corneal haze in eyes of the SMILE Xtra group, and this was reflected on the significant improvement of UDVA and CDVA. However, corneal densitometry decreased over time but did not reach its baseline levels. Ng et al. [24] reported a high level of refractive predictability. At 6 months, 89% of their SMILE Xtra eyes were within ± 0.50 D from target and 100% were within ± 1.00 D. This was comparable to our results with 87% of eyes were within ± 0.50 D and 100% of eyes were within ± 1.00 D. In our study, no eyes lost more than 1 line of CDVA which was the same as SMILE Xtra series reported by Ganesh et al. [23] and Ng et al. [24].

Regarding CCT, the SMILE Xtra group showed statistically significant decrease from 1 to 3 months which increased again by 6 months and stabilized during the rest of the follow-up period. This change can be explained by the compaction of the corneal stroma because of collagen cross-linking. At 24 months, the SMILE group had significantly thicker CCT, which can be explained by the lower myopic error corrected plus the compaction of stroma due to CXL as well as having thinner mean CCT from the start. Regarding ECD, both groups proved to be safe with no significant changes in the endothelial cell count. This agreed with the previous reports, denoting no significant change in the ECD before and after SMILE Xtra [23] and after SMILE. [34] Regarding keratometry, the SMILE Xtra group showed flattening of the cornea that continued throughout the first 6 months, then relatively stabilized. This might be explained by the flattening effect of the CXL on the cornea which could be desirable in keratoconus but could be a cause of regression when used in normal corneas undergoing SMILE. In the current study, we used half the duration of the standard CXL treatment to avoid too much flattening effect on the cornea.

Regarding corneal biomechanics, CRF was significantly higher in the SMILE Xtra group and remained stable over the 24 months of follow-up. This is a strong proof for the benefit of the simultaneous use of corneal collagen cross-linking with SMILE to strengthen the weakened cornea at risk of ectasia. The lack of actual measurements for the corneal biomechanics was a major disadvantage in the reported studies that used SMILE Xtra for prophylaxis against ectasia in high-risk eyes [23, 24].

The advantages of the current study are the comparative nature with a control group of SMILE cases, relatively good follow-up period of 24 months, and the actual measurement of corneal biomechanics to prove the efficacy of the simultaneous use of CXL with SMILE. To our knowledge, this is the longest published follow-up period for the use of SMILE Xtra for prophylactic treatment of corneal ectasia in high-risk patients. Among the limitations of our study were the requirement of more included cases and the retrospective nature of the study. Also, a longer duration of follow-up to record the long-term effects, e.g., regression and presence of ectasia might be required. Two years follow-up looks good but still not enough to show ectasia results. Because in most cases, ectasia appears after 3 years, so longer-term follow-up studies are needed.

In conclusion, combining corneal cross-linking with SMILE procedure (SMILE Xtra) is a promising tool to prevent ectasia in high-risk patients. It is a safe and simple procedure that can be offered to patients undergoing SMILE with risk for ectasia.

## Figures and Tables

**Table 1:** Preoperative characteristics of the included eyes (60 eyes of 30 patients divided equally into two groups).

	SMILE Xtra (*n*=30) (mean ± SD)	SMILE (*n*=30) (mean ± SD)	*P* value
Male : female	10 : 5	8 : 7	0.456^a^
Age (years)	24.3 ± 5.9	25.2 ± 5.1	0.640^b^
MRSE (D)	−8.6 ± 1.1	−8.2 ± 1.2	0.411^b^
CDVA (logMAR)	0.048 ± 0.06	0.042 ± 0.07	0.341^b^
CCT (microns)	495.1 ± 22.6	498.4 ± 21.1	0.245^b^
K readings (D)	45.1 ± 1.5	44.6 ± 1.2	0.228^b^
ECD (cells/mm^2^)	2816 ± 288	2840 ± 278	0.755^b^
CRF	10.42 ± 1.68	10.82 ± 1.69	0.287^b^
Densitometry	17.31 ± 1.90	16.51 ± 1.71	0.299^b^

MRSE: manifest refraction spherical equivalent; K: keratometry; CDVA: corrected distance visual acuity; CCT: central corneal thickness; ECD: endothelial cell density; CRF: corneal resistance factor. ^a^Chi-squared test; ^b^independent-samples *t*-test.

**Table 2:** Visual acuity and refraction along the postoperative follow-up period.

	1 month	3 months	6 months	12 months	24 months
SMILE Xtra: UDVA (logMAR)	0.068 ± 0.08	0.033 ± 0.07	0.031 ± 0.06	0.030 ± 0.06	0.031 ± 0.06
SMILE: UDVA (logMAR)	0.041 ± 0.07	0.031 ± 0.06	0.029 ± 0.06	0.028 ± 0.06	0.028 ± 0.05
*P* value	0.003^*∗*^	0.124	0.122	0.130	0.144
SMILE Xtra: CDVA (logMAR)	0.021 ± 0.09	−0.028 ± 0.07	−0.042 ± 0.06	−0.051 ± 0.05	−0.055 ± 0.05
SMILE: CDVA (logMAR)	0.002 ± 0.07	−0.038 ± 0.06	−0.048 ± 0.06	−0.055 ± 0.06	−0.057 ± 0.05
*P* value	<0.001^*∗*^	0.031^*∗*^	0.171	0.190	0.272
SMILE Xtra: MRSE (D)	−0.12 ± 0.23	−0.16 ± 0.25	−0.19 ± 0.21	−0.20 ± 0.21	−0.18 ± 0.19
SMILE: MRSE (D)	−0.18 ± 0.19	−0.20 ± 0.22	−0.21 ± 0.21	−0.22 ± 0.20	−0.19 ± 0.18
*P* value	<0.001^*∗*^	0.038^*∗*^	0.267	0.278	0.361

Values are expressed as mean ± SD. SD: standard deviation; UDVA: uncorrected distance visual acuity; CDVA: corrected distance visual acuity; *p* value compares mean of SMILE Xtra vs. SMILE using independent-samples *t*-test. ^*∗*^Statistically significant.

**Table 3:** Average keratometry readings, central corneal thickness, and endothelial cell count along the postoperative follow-up period.

	1 month	3 months	6 months	12 months	24 months
SMILE Xtra: K readings (D)	36.4 ± 1.8	36.3 ± 1.7	36.2 ± 1.6	36.1 ± 1.6	36.1 ± 1.7
SMILE: K readings (D)	36.2 ± 1.6	36.4 ± 1.8	36.3 ± 1.6	36.4 ± 1.7	36.3 ± 1.8
*P* value	0.388	0.585	0.457	0.399	0.416
SMILE Xtra: CCT (microns)	408.1 ± 42.6	402.3 ± 41.6	405.1 ± 42.2	407.3 ± 40.6	407.3 ± 41.6
SMILE: CCT (microns)	412.2 ± 39.8	410.6 ± 40.8	412.5 ± 39.6	411.2 ± 38.8	413.2 ± 39.5
*P* value	0.179	0.265	0.048^*∗*^	0.066	0.041^*∗*^
SMILE Xtra: ECD (cells/mm^2^)	2776 ± 318	2775 ± 298	2780 ± 295	2776 ± 310	2790 ± 300
SMILE: ECD (cells/mm^2^)	2815 ± 288	2818 ± 278	2813 ± 286	2819 ± 280	2810 ± 298
*P* value	0.654	0.644	0.599	0.623	0.610

Values are expressed as mean ± SD. D: diopters; K: keratometry; CCT: central corneal thickness; ECD: endothelial cell density; *p* value compares mean of SMILE Xtra vs. SMILE using independent-samples *t*-test. ^*∗*^Statistically significant.

**Table 4:** Corneal resistance factor and corneal densitometry along the postoperative follow-up period.

	1 month	3 months	6 months	12 months	24 months
SMILE Xtra: CRF	9.72 ± 1.55	9.76 ± 1.63	9.77 ± 1.68	9.74 ± 1.77	9.72 ± 1.66
SMILE: CRF	9.12 ± 1.79	9.15 ± 1.65	9.20 ± 1.69	9.19 ± 1.70	9.22 ± 1.66
*P* value	0.001^*∗*^	0.001^*∗*^	0.001^*∗*^	0.001^*∗*^	0.001^*∗*^
SMILE Xtra: Densitometry	24.11 ± 2.55	22.32 ± 2.14	20.16 ± 1.91	18.61 ± 1.85	18.42 ± 1.88
SMILE: Densitometry	17.41 ± 1.61	17.01 ± 1.66	16.91 ± 1.70	16.81 ± 1.61	16.80 ± 1.65
*P* value	0.001^*∗*^	0.001^*∗*^	0.001^*∗*^	0.001^*∗*^	0.001^*∗*^

Values are expressed as mean ± SD. SD: standard deviation; CRF: corneal resistance factor; *p* value compares mean of SMILE Xtra vs. SMILE using independent-samples *t*-test. ^*∗*^Statistically significant.

## Data Availability

The data used to support the findings of this study are available from the corresponding author upon request.

## Abbreviations

SMILE: Small incision lenticule extraction
PRK: Photorefractive keratectomy
LASIK: Laser in situ keratomileusis
CXL: Corneal collagen cross-linking
UDVA: Uncorrected distance visual acuity
CDVA: Corrected distance visual acuity
CCT: Central corneal thickness
ECD: Endothelial cell density
MRSE: Manifest refraction spherical equivalent
UV-A: Ultraviolet-A
CRF: Corneal resistance factor.
